# Cohort study protocol to characterize the incidence and severity of neuropathic pain in patients with severe acute respiratory syndrome coronavirus 2 infection

**DOI:** 10.1097/PR9.0000000000000925

**Published:** 2021-04-20

**Authors:** Chioma U. Odozor, Kristen Roles, Carrie Burk, Thomas Kannampallil, David B. Clifford, Jay F. Piccirillo, Simon Haroutounian

**Affiliations:** a Washington University School of Medicine, St. Louis, MO, USA; b Department of Anesthesiology, Washington University School of Medicine, St. Louis, MO, USA; c Institute for Informatics, Washington University School of Medicine, St. Louis, MO, USA; d Department of Neurology, Washington University School of Medicine, St. Louis, MO, USA; e Department of Otolaryngology-Head & Neck Surgery, Washington University School of Medicine, St. Louis, MO, USA

**Keywords:** Neuropathic pain, SARS-CoV-2, COVID-19, Neuropathy, Coronavirus

## Abstract

**Introduction and Objectives:**

The coronavirus disease 2019 (COVID-19) pandemic has resulted in patients experiencing symptoms that include neurological dysfunction. As many viral infections are associated with neuropathy, the aim of the study is to characterize the incidence and severity of neuropathic pain in patients with COVID-19.

**Methods:**

A cohort study will be conducted in adult (≥18 years) patients who were tested for severe acute respiratory syndrome coronavirus 2 (SARS-CoV-2) at Washington University/Barnes-Jewish Hospital. Participants who are deceased, with incomplete test results, or who cannot be contacted will be excluded. Approximately 1320 participants will be recruited in a 1:2 ratio of those with a positive-to-negative SARS-CoV-2 test result. Each participant will be invited to complete a survey to assess their symptoms related to neuropathy, 30 to 90 days after their initial SARS-CoV-2 test. Survey responses, demographics, and clinical data from the electronic health record will be used for analysis. The primary outcome is the incidence of new symptoms of neuropathic pain. The self-reported DN4 and Neuropathic Pain Symptom Inventory questionnaires (Appendix 1, http://links.lww.com/PR9/A103) will be used for neuropathic pain screening and severity assessment, respectively. Exploratory analyses will be performed to investigate other potential clinical endpoints and trends.

**Results/Conclusion:**

Similar to previous coronavirus infections, an increased incidence of new-onset neuropathic pain after COVID-19 disease is expected, along with an increase in the severity experienced by patients with COVID-19 with pre-existing chronic pain. Comprehensive understanding of how COVID-19 affects the nervous system can provide a better framework for managing pain in this disease.

## Abstract 2

Supplemental Digital Content is Available in the Text.

In this protocol for a cohort study, an approach to characterize new-onset or worsening neuropathic pain in patients with COVID-19 is described.

## 1. Introduction

December 2019 witnessed the identification of a novel coronavirus, severe acute respiratory syndrome coronavirus 2 (SARS-CoV-2), in Wuhan, China, which has escalated to a global pandemic with a 2.6% mortality rate. As of November 1, 2020, more than 47 million confirmed cases have been reported worldwide.^22^ Coronavirus disease 2019 (COVID-19) syndrome is the disease caused by SARS-CoV-2 infection, with common symptoms including cough, headache, fever, dyspnea, and fatigue to more severe sequelae such as severe respiratory distress, acute cardiac injury, thrombosis, and death.^8^

Severe acute respiratory syndrome coronavirus 2 is the seventh identified coronavirus capable of infecting humans^24^ and shares many molecular and clinical similarities to the other betacoronaviruses, such as MERS-CoV and SARS-CoV, which caused Middle East respiratory syndrome (MERS) and severe acute respiratory syndrome (SARS), respectively.^16,23,25^ Emerging reports of changes to smell and taste perception (anosmia, hyposmia, ageusia, or dysgeusia)^13,14^ in patients with COVID-19, even before or without the manifestation of other symptoms related to SARS-CoV-2 infection,^6^ suggest a possible sensory dysfunction in the course of this disease.

Both MERS and SARS, along with other viruses such HIV, varicella zoster virus, and hepatitis C, have been associated with peripheral neuropathies.^5,9–11,15,19,21^ There are several published case series, small-scale retrospective observational studies, and anecdotal evidence of neuropathic pain and other neurological symptoms in patients with confirmed COVID-19.^12,14,17,20^ However, it is unclear whether SARS-CoV-2 infection and subsequent COVID-19 disease are associated with an increased risk of painful peripheral neuropathy or cranial nerve dysfunction. Timely and accurate diagnosis of neuropathy and neuropathic pain can allow early intervention and possible mitigation of long-term symptoms and quality of life impairment. The goal of this cohort study will be to determine the incidence and characteristics of neuropathic pain symptoms and cranial nerve dysfunction associated with COVID-19.

## 2. Methods

### 2.1. Study design and participants

The protocol for this study was approved by the Institutional Review Board (IRB) at the Washington University School of Medicine in St. Louis (IRB# 202004043). We will conduct a cohort study of individuals who were tested for SARS-CoV-2, using a reverse transcriptase polymerase chain reaction (RT-PCR) testing. Participants will be divided into 2 cohorts based on either a positive (CV19+) or negative (CV19−) result on their SARS-CoV-2 RT-PCR test.

Participants will be recruited from 3 sites that were part of the same academic medical center in Missouri, USA, as identified through the Epic electronic health record system (Verona, WI). The study sites include the Barnes-Jewish Hospital, the Washington University School of Medicine, and the St. Louis Children's Hospital. All participants will be at least 18 years old and would have presented for SARS-CoV-2 testing for any indication (eg, symptomatic presentation, possible exposure, or perioperative screening) to one of the clinics or hospitals associated with the study sites on or after March 16, 2020. Participants will be excluded if they were younger than 18 years old, known to be deceased at the time of sending study surveys, or do not have available RT-PCR test results or contact information on their patient record. Study participants will be provided with a consent information sheet along with the study survey and will indicate their consent by completing the web-based or mail-based survey (Appendix 1, available at http://links.lww.com/PR9/A103).

### 2.2. Data collection

Demographic and clinical data including age, sex, race, ethnicity, marital status, medical history, tobacco and alcohol use, comorbidities, medications, associated symptoms (eg, fever, cough, pneumonia, anosmia, or ageusia), available influenza test results, and COVID-19–related hospitalization details (if hospitalized) will be extracted from the patient record. COVID-19 disease severity will be assessed based on the level of care (hospital admission and intensive care unit admission), length of hospitalization, time to intubation from disease onset, if known, and mechanical ventilation requirement and settings.

An email with a link to a secure web-based survey on the REDCap platform^7^ will be sent to all participants approximately 30 to 90 days from the time of their SARS-CoV-2 RT-PCR test results. As a follow-up, participants who do not respond to 2 online survey invitations may be contacted through telephone and invited to participate in the web-based survey or provide a mailing address to send a paper copy of the survey, along with a consent form, by postal mail with a prepaid return envelope.

The survey includes questions on patient-reported symptoms of peripheral neuropathy or cranial nerve dysfunction at 3 time points: at the time of initial symptoms (or SARS-CoV-2 testing, if asymptomatic), 1 to 2 weeks after symptom manifestation or testing, and at the time of questionnaire completion (Appendix 1, available at http://links.lww.com/PR9/A103). Data will also be collected on previous chronic pain or neuropathic conditions. For those reporting symptoms of neuropathy or nerve dysfunction, a branching logic will be used to (1) assess the anatomical distribution of pain or neuropathy on a body diagram, (2) complete the 7-item self-report DN4 neuropathic pain screening questionnaire,^1^ and (3) complete the Neuropathic Pain Symptom Inventory (NPSI).^2^

### 2.3. Outcomes

The primary outcome of this study is the incidence of new symptoms of peripheral neuropathy, between the CV19+ and CV19− patient cohorts. DN4 and NPSI scores will be used to screen for neuropathic pain and determining its severity, respectively (Appendix 1, available at http://links.lww.com/PR9/A103). Additional outcomes include symptoms of motor neuropathy and cranial nerve I to XII dysfunction and change in severity of pre-existing chronic pain or neuropathy. The variety of symptoms associated with new or worsened neuropathy will be qualitatively compared between the 2 cohorts.

### 2.4. Statistical analysis

Continuous data will be expressed as mean (standard deviation) or median (interquartile range) values, as appropriate, and categorical variables will be expressed as frequencies, n (%). Differences in symptoms and baseline characteristics between the 2 cohorts will be reported using effect size metrics and 95% confidence intervals. Demographics of survey responders and nonresponders will be compared to determine the risk of responder bias. The overall incidence of new symptoms of peripheral neuropathy or cranial nerve dysfunction, based on the survey, will be compared between the 2 cohorts using the Fisher exact test. Continuous data will be assessed for normality using the Shapiro–Wilk test, and nonparametric and parametric data will be analyzed using a Mann–Whitney *U* test or two-sample *t* test, respectively, where applicable.

Multivariable logistic regression with composite outcome of new peripheral neuropathy, adjusted for demographic factors and comorbidities, will be performed. Bivariate analyses of clinical characteristics will be performed to identify associations with an incidence of new peripheral neuropathy, which will therefore be included in the multivariable logistic regression model. Assuming a 1% incidence of new neuropathic pain in the CV19− cohort, to detect an incidence of 3.5% or more of peripheral neuropathy in the CV19+ cohort with 80% power and α = 0.05, the study will require 1320 participants overall in a 1:2 ratio of CV19+ to CV19− subjects (ie, 440 CV19+ and 880 CV19−).

As a secondary outcome, the incidence of neuropathic pain based on a DN4 score ≥4 will be compared between the 2 cohorts using the χ^2^ test. Finally, a *t* test will be used to compare the severity of neuropathy from the total NPSI score between the CV19+ and CV19− cohorts.

As the study cohorts are defined by the SARS-CoV-2 RT-PCR test result, and not an initial clinical presentation of COVID-19 disease, 2 sensitivity analyses will be conducted based on (1) patients' self-reported symptoms as a reason for testing and (2) clinical diagnoses of COVID-19 in the Epic electronic health record, pending complete data availability.

All statistical analyses will be performed using R software version 4.0.1. A *P* value of <0.05 is the threshold for statistical significance for all analyses.

## 3. Discussion

This cohort study aims to characterize neuropathic pain symptoms in patients with SARS-CoV-2 infection. Study participants in the CV19− cohort will serve as a comparator to elucidate the differences in severity and incidence of peripheral neuropathy among patients with and without a positive SARS-CoV-2 RT-PCR test. The CV19− cohort will be selected among patients from the same geographical area and are tested for SARS-CoV-2, to account for similar circumstances that could have resulted in a new onset of neuropathy symptoms. Neurological symptoms will be compared between the cohorts and then discussed in the context of current literature on neuropathic pain after other viral infections.

Sensory and motor peripheral neuropathies after MERS and SARS infections have been extensively reported.^11,21^ In both cases, the onset of neurological symptoms occurred 2 to 3 weeks after the initial viral infection and involved varying complications, such as limb weakness, hyporeflexia, and hypoesthesia in 2 or all 4 limbs. In our study, a time frame of 1 to 3 months was selected to account for the possible delay in symptom manifestation after the initial acute nerve insult. In a systematic review of patients with COVID-19 who were admitted to a Spanish hospital, 57.4% of patients experienced neurological symptoms^17^; this prevalence is similar to a retrospective study in Wuhan, China, where 36.4% of the study population experienced neurological symptoms such as dizziness (16.8%), headache (13.1%), impaired consciousness (7.5%), skeletal muscle injury (10.1%), and peripheral nervous system symptoms, such as sensory impairment and nerve pain (8.9%).^14^ In the study by Romero-Sanchez et al. (2020), viral RNA could not be detected in any of the cerebrospinal fluid samples. Although the exact mechanism for how SARS-CoV-2 affects the peripheral nervous system is not yet known, Shiers et al. ^18^ found that a subset of human dorsal root ganglion neurons were nociceptors that expressed the mRNA of the SARS-CoV-2 receptor, ACE2. They suggested that the nociceptors could form free nerve endings on the skin and luminal organs, serving as a route for SARS-CoV-2 infection. Results from our study will be discussed and compared in the context of the published literature.

The study is limited by the lack of complete neurological examinations by an independent neurologist for each participant. For example, symptoms of blurred vision, diplopia, or vision loss could be due to cranial nerves II, III, IV, and/or VI; however, without an in-person neurological examination it is difficult to further localize the ocular lesion. Clinical signs of peripheral neuropathy, muscle pain, and cranial nerve dysfunction will be self-reported without necessarily having an accompanying clinical diagnosis on their medical record. However, a strength of this study is the use of DN4 and NPSI scores as 2 individually validated questionnaires to screen for neuropathic pain.^1,2^ There is a potential risk of recall bias because some parts of the questionnaire ask participants about their symptoms at the time of testing or symptom onset. However, as the time frame for recall is relatively short and applied to all study participants, this potential for bias is likely to be low and balanced between the 2 study cohorts. Finally, the possibility of false negatives from RT-PCR testing for SARS-CoV-2 may mean that some of the participants in the CV19− cohort should have been in the CV19+ cohort. This potential confounder in our study may affect the findings and make it more difficult to detect a true difference between the 2 cohorts. The early detection of viral-induced neuropathies is important to treat and prevent more severe complications after infection and to aid in rational drug design approaches. The delay in reporting peripheral neuropathy in people with HIV led to millions being underdiagnosed or undertreated during the AIDS epidemic in the 1980s.^3,4^ Should there be a significant increase in neuropathy after SARS-CoV-2 infection, our findings may add to the literature supporting a role for the nervous system in the pathogenesis of COVID-19 disease.

## Disclosures

The authors have no conflicts of interest to declare.

## Appendix A. Supplemental digital content

Supplemental digital content associated with this article can be found online at http://links.lww.com/PR9/A103.
